# Temporary Hospital Staff Appointments

**Published:** 1918-02-23

**Authors:** 


### TEMPORARY HOSPITAL STAFF APPOINTMENTS.

The movement of time, alike in war as in peace, brings changes in all departments, and, among others, in the staffs of our hospitals. Indeed, the war has multiplied these changes, for hospital staffs, even more than the general population, have been invaded by the claims and fatalities of the conflict. The vacancies so created have to be filled up in quite exceptional circumstances. There are, it is to be noted, two entirely different positions. In the first place are vacancies which are nominal or formal rather than substantial. The physician or surgeon is absent from his hospital service because he is serving with the R.A.M.C., or is in some other capacity doing Government work. In these instances no one would propose to deprive him of his hospital appointments. He has won these appointments by years of hard and unremunerative labour, and he has put them on one side as a sacrifice to the national cause. It would be eminently unjust to supplant him in his hospital position and to leave him to find, on returning home, that his place had been taken by one of his *confrères*. Hence such a position has been met, we believe uniformly, by the election of a temporary officer, and members of the profession thus called upon to act as substitutes have willingly accepted this arrangement.

The position, however, is not quite so simple when the vacancy to be filled is substantial, as happens by death or resignation. Here is no holder of an office whose interests have to be considered in view of his absence from hospital duty on patriotic service. The vacancy is real, and someone must be appointed to fill it. But here, also, the custom seems to have been to make a temporary election for the duration of the war. On the whole, this plan seems to be the best, both in the interests of the hospitals and in those of individuals. It is impossible in existing circumstances to obtain for such vacancies the number of candidates which could be expected in times of peace. So many men are absent on military duty that necessarily the choice is a restricted one. In other words, the hospital does not get the opportunity which in normal times it would enjoy. This opportunity will return at or soon after the declaration of peace, and hence sound policy would seem to dictate an arrangement by which the work can be carried on in the meantime, leaving the final judgment for an occasion when a wider choice will become available.

What is best for the institution is necessarily the first consideration. But, this granted, it is fair to bear in mind the interests of individuals, and more especially of those who are called by stern duty out of the chance of appointments at home. Permanently to fill up vacancies for which some of these men would in normal circumstances have been candidates is to penalise those who are already sacrificing much in the country's interest. It would seem to be a manifest duty to avoid such a position unless the general interest renders it imperative. But, as we have here tried to show, no such necessity exists. On the contrary, the welfare of the hospitals seems to harmonise with the consideration due to individual interests, and we therefore express our entire sympathy with the practice which has been generally adopted and by which vacancies on hospital staffs are during the duration of the war met by the election of temporary officers.

